# Polymerized Laminin-521: A Feasible Substrate for Expanding Induced Pluripotent Stem Cells at a Low Protein Concentration

**DOI:** 10.3390/cells11243955

**Published:** 2022-12-07

**Authors:** Fernanda C. P. Mesquita, Eliel S. Leite, Jacquelynn Morrissey, Catarina Freitas, Tatiana Coelho-Sampaio, Camila Hochman-Mendez

**Affiliations:** 1 Department of Regenerative Medicine Research, The Texas Heart Institute, 6770 Bertner Avenue, MC 1-135, Houston, TX 77030, USA; 2 Institute of Biomedical Sciences, Federal University of Rio de Janeiro, Av. Carlos Chagas Filho B1-011, 373, Rio de Janeiro 21941-902, Brazil

**Keywords:** polylaminin, laminin 521, iPSC, cell expansion, pluripotency

## Abstract

Laminins (LNs) play a central role in the self-assembly and maintenance of basement membranes and are involved in critical interactions between cells and other extracellular matrix (ECM) proteins. Among the defined, xeno-free ECM culture matrices, LNs—namely LN521—have emerged as promising coating systems for the large-scale expansion of induced pluripotent stem cells (iPSCs). The biologic activity of LNs is enhanced by their acidification-induced self-polymerization into a cell-associated network called polylaminin (polyLN), which can recapitulate the native-like polymeric array in a cell-free system. Here, we show for the first time to our knowledge that polyLN521 displays a native-like hexagonal-like structure and that, at basal and low concentrations, it permits the large-scale expansion of human iPSCs. Human iPSCs expanded with polyLN521 maintained the pluripotent state and showed no impairment of karyotype stability or telomere length. These results suggest that low-concentration polyLN521 is a stable and cost-effective coating for large-scale iPSC expansion.

## 1. Introduction

During the last two decades, pluripotent stem cell (PSC) research has blossomed into an exciting field with great potential for application in the areas of regenerative medicine, disease modeling, drug discovery, and human developmental biology [1]. Although the capacity of PSCs to differentiate makes them appealing for research applications, their plasticity poses challenges to ensuring that, upon expansion, cells remain stable and maintain their pluripotent capacity for differentiation [2]. Substantial effort has been dedicated to optimizing cell culture conditions that maintain cell stability and decrease heterogeneity within batch populations and between culture batches [3]. One of the most important advances has been the use of defined media formulations in combination with a feeder-free coating system, such as Matrigel [4,5]. However, Matrigel exhibits batch-to-batch variability and is xenogeneic, complicating its widespread use for regenerative medicine applications [6]. As alternatives, the individual proteins laminin (LN), vitronectin, and fibronectin, as well as synthetic polymers, have been described as viable, well-defined coating options that minimize batch-to-batch variability during PSC culture expansion [7,8,9].

Laminin is the primary component of Matrigel and is responsible for its biologic activity in various scenarios such as capillary-like tubulogenesis and mammary acini morphogenesis in vitro [10,11,12]. Because of the critical role of LN isoforms in cell proliferation and differentiation during embryogenesis [13], they have been investigated for their suitability in feeder-free culture systems [14]. In particular, the LN isoforms LN511 and LN521 have been shown to be efficacious in generating and maintaining PSCs under these chemically-defined conditions [15,16]. The cells cultured in LN-based systems have shown evidence of efficient attachment, long-term self-renewal, pluripotent marker expression, normal karyotypes, and differentiation capabilities in vitro and in vivo [17,18]. Overall, these recent advances have led to the establishment of recombinant LN as a desirable coating for the cultivation of PSCs. Nonetheless, the scaled-up expansion of PSCs in the multibillion range (i.e., the amount needed to populate whole organs) requires technical improvements to circumvent the immense cost of coating the cell-adhesion surface of a large-scale reactor with recombinant proteins. A critical step towards this goal is to optimize the amount of recombinant LN required for PSC cultivation.

Recently, decreasing the concentration of LN511 for PSC cultivation was shown to progressively reduce colony size and impair expansion capability [19]. The loss of efficacy observed at a lower concentration of LN511 may be related to the formation of a discontinuous coating that is unable to support the uniform distribution of cells on the substrate. In line with this view, LN extracted from Engelbreth-Holm-Swarm (EHS) murine tumor that was adsorbed onto culture substrates showed a tendency to form large aggregates on top of which neurons preferentially adhered [20]. On the other hand, polylaminin (polyLN), a biomimetic polymer of laminin, showed a more homogenous distribution across the substrate [21]. Atomic force microscopy studies further demonstrated that, in contrast to ordinary LN, an adsorbed layer of polyLN had a flat morphology and a fairly homogeneous height [22]. Polylaminin-based substrates have been shown to be biocompatible for the cultivation of several cell types, including adipose tissue–derived mesenchymal cells, Schwann cells, neurons, and epithelial cells such as follicular thyroid cells, keratinocytes, and endothelial cells [21,23,24,25]. Moreover, polyLN has been used to establish colonies of mammary epithelial cells lacking the dystroglycan receptor and restore their ability to produce milk in vitro [26].

In this study, we compared the growth and phenotype of human iPSCs (hiPSCs) cultivated on Matrigel (the gold standard), recombinant LN521, or polyLN521 at normal basal (5 µg/mL) and low (0.5 µg/mL) concentrations. We hypothesized that polyLN521 may be a simple, robust alternative for maintaining colony integrity upon dilution. Our findings provide evidence that polyLN521 improved hiPSC adhesion, proliferation, and maintained the pluripotent state even at the low concentration.

## 2. Materials and Methods

### 2.1. Microscopy

Human recombinant LN521 (Biolamina, Sundbyberg, Sweden) was diluted to 20 µg/mL in acidic buffer (20 mM sodium acetate, pH 4.0) containing 1 mM CaCl_2_. Drops containing 10 μL were placed on glass coverslips and incubated for 12 h, after which they were fixed in either 4% paraformaldehyde for immunolabeling or in Karnowsky reagent (4% paraformaldehyde and 0.5% glutaraldehyde in 0.1 M cacodylate buffer, pH 7.2) for 2 h for scanning electron microscopy (SEM). For immunolabeling, samples were incubated with rabbit polyclonal anti-LM antibody (1:50; Millipore-Sigma, St. Louis, MO, USA) and then with Alexa Fluor 555 anti-rabbit IgG (1:300, Thermo Fisher Scientific, Waltham, MA, USA). Confocal fluorescence images were obtained using a Zeiss Elyra PS1. For SEM, dehydrated samples dried with critical point drying were coated with a thin layer of gold sputter and visualized by using a JEOL JSM6300 scanning electron microscope. For transmission electron microscopy (TEM), 3 μL of polyLN521 were added to 300 mesh Cu-grids (Ted Pella Inc., Redding, CA, USA) that were previously glow-discharged for 180 s at 15 mA (Ted Pella, PELCO easiGlow™). After 30 min, the excess liquid was removed, and 1% uranyl acetate was added to samples for contrasting. Samples were analyzed using a 120-kV transmission electron microscope (Hitachi HT 7800, Japan).

### 2.2. Coating Preparation

Matrigel^®^ Human Embryonic Stem Cell (hESC)-qualified Matrix (Corning Inc., Corning, NY, USA) was diluted to 100× (basal-Matrigel) and 1000× (low-Matrigel) in cold sterile phosphate-buffered saline (PBS), and 1 mL of solution was added to a 35-mm^2^ plate and maintained for 1 h at 37 °C before the cells were plated. Human recombinant LN521 was diluted in sterile PBS (pH 7.0) at final concentrations of 5 µg/mL (basal-LN521) and 0.5 µg/mL (low-LN521). For polyLN521, human recombinant LN521 (100 µg/mL) was diluted in cold, sterile acid buffer solution (20 mM sodium acetate, 1 mM CaCl_2_, pH 4.0) at final concentrations of 5 µg/mL (basal-polyLN521) and 0.5 µg/mL (low-polyLN521). One mL each of LN521 or polyLN521 was added to a 35-mm^2^ plate and maintained overnight at 37 °C. The next day, the plates were washed 3 times with PBS before the cells were plated.

### 2.3. Cell Expansion

The hiPSC lines (SCVI20 and SCVI274) used in this study were kindly donated by the Stanford Cardiovascular Institute Biobank (Stanford, CA, USA). Cells were cultured and maintained in a feeder-free system of hESC-qualified Matrigel™ and StemFlex™ Medium (Gibco, Waltham, MA, USA) under standard culture conditions (37 °C and 5% CO_2_). Briefly, 100-mm petri dishes were coated with Matrigel for at least 1 h at 37 °C, followed by the plating of 1 × 10^5^ cells in StemFlex™ media supplemented with ROCK inhibitor Y-27632 (10 µM, STEMCELL Technologies Inc., Cambridge, MA, USA) for 24 h. The medium was changed daily, and the cells were passaged using the cell dissociation recombinant enzymatic solution TrypLE™ Express (Gibco). Next, 0.2 × 10^6^ hiPSCs were plated under the basal coating conditions (basal-Matrigel, basal-LN521, and basal-polyLN521) and expanded for 3 passages. After that, 0.2 × 10^6^ hiPSCs were plated for each experimental condition (basal-Matrigel, low-Matrigel, basal-LN521, low-LN521, basal-polyLN521, and low-polyLN521) and maintained as described above for 4 days. Cell viability was determined by staining with Trypan blue. Live and dead cells were counted using a hemocytometer.

### 2.4. Colony Area

Ten random bright-field images were captured using an inverted microscope (Leica DMI3000B), and colony area was measured from the second to the fourth day after plating (*n* = 4) using ImageJ (National Institutes of Health, Bethesda, MD, USA). Individual colonies were identified by assessing colony brightness, density, and border characteristics. Using the “Straight” tool function, the scale bar was measured, and calibration and conversion of the unit pixel pattern to µm^2^ was carried out. Then, using the “Freehand selections” tool, each colony was traced along its border, and the area was calculated.

### 2.5. DNA Extraction

Genomic DNA was extracted from cell culture samples using the Blood & Cell Culture DNA Mini Kit (Qiagen, Hilden, Germany) according to the manufacturer’s instructions. Briefly, cells were lysed and digested with protease, and DNA was isolated from other cellular components using a selective resin column. Purified DNA was eluted, dissolved in TE buffer (pH 8.0), and quantified using a NanoDrop™ Lite Spectrophotometer (Thermo Scientific™, Wilmington, DE, USA).

### 2.6. Genetic Analysis of hiPSCs

The genomic stability of the cells was evaluated using the hPSC Genetic Analysis Kit (STEMCELL Technologies Inc., Vancouver, Canada). Briefly, genomic DNA was extracted from cells, and chromosomal quantitative PCR (qPCR) was performed using the QuantStudio™6 Flex Real Time PCR System (Applied Biosystems™, Singapore) according to the manufacturer’s instructions. The qPCR data were analyzed using the Genetic Analysis App (https://shiny.stemcell.com/ShinyApps/psc_genetic_analysis_app/ [accessed on 28 February 2022]).

### 2.7. Telomere Length

Telomere length in hiPSCs was evaluated using the Absolute Human Telomere Length Quantification qPCR Assay Kit (ScienCell Research Laboratories, Carlsbad, CA, USA). The 2X GoldNStart TaqGreen qPCR master mix was used for amplification, and genomic DNA was analyzed at a concentration of 0.25 ng/μL. The telomere length of each sample was determined using the ΔΔCq method.

### 2.8. Gene Expression

RNA was extracted from samples using the miRNeasy Mini Kit (Qiagen), and cDNA was produced using the High-Capacity cDNA Reverse Transcription Kit (Applied Biosystems, Waltham, MA, USA). For PCR amplification, Platinum™ Taq Green Hot Start DNA Polymerase (Invitrogen™, Vilnus, Lithuania) was used. PCR products were analyzed by electrophoresis in agarose gels using GAPDH as the housekeeping gene. Gene expression profiles were determined using the SYBR™ Select Master Mix (Applied Biosystems, Carlsbad, CA, USA). Gene expression was analyzed using the ΔCt method, in which expression of the gene was compared to expression of the housekeeping gene GAPDH. Basal-Matrigel at day 2 was used as a reference sample for all analyses. A list of all primers is provided in Supplementary Table S1.

### 2.9. Flow Cytometry

Human iPSCs were harvested using TrypLE™ Express (Gibco). For nuclear staining, cells were fixed and permeabilized using the BD Cytofix/Cytoperm™ Kit (BD Biosciences, Franklin Lakes, NJ, USA) according to the manufacturer’s instructions. Briefly, cells were resuspended and incubated in 250 μL of BD Cytofix/Cytoperm solution for 20 minutes at 4 °C. After centrifugation (600× *g* for 5 min), the cells were resuspended in BD Perm/Wash buffer for 30 min at 4 °C. Cells were stained with Alexa Fluor^®^ 488 mouse anti-Oct3/4, Phycoerythrin (PE) mouse anti-human Nanog, or Alexa Fluor^®^ 647 mouse anti-Sox2. For membrane markers, the cells were stained directly with PE mouse anti-SSEA-4 or fluorescein isothiocyanate (FITC) mouse anti-human TRA-1-60 (BD Biosciences). Isotypes were used as negative controls. Samples were analyzed using BD LSRFortessa and FlowJo v10.7.1 software.

### 2.10. Differentiation Potential of hiPSCs

Spontaneous differentiation of hiPSCs into the three embryonic germ layers was induced as previously described [16]. Briefly, the hiPSCs were detached from the cell culture plates using a cell scraper (TWD TradeWinds Inc., Pleasant Prairie, WI, USA) and cultured in ultra-low attachment 6-well plates (Corning) to form embryoid bodies (EBs). The EBs were cultured in suspension for 5 days at 37 °C and 5% CO_2_ with basal medium containing the following: DMEM/F-12, GlutaMAX™ supplement, 20% KnockOut™ Serum Replacement, 1% MEM Non-essential Amino Acids, and 55 mM^2^-mercaptoethanol (Gibco). Then, EBs were transferred to adherent plates and cultured for an additional 5 days in the basal medium. Gene expression of the differentiated cells was analyzed as described above.

### 2.11. Large-Scale Expansion of hiPSCs

Human iPSCs were expanded on a large scale in the Quantum Expansion System (QES, Terumo BCT) using a modified protocol [16,27] in which polyLN521 is used as the coating substrate. Briefly, the hollow-fiber surfaces were coated overnight with a basal (5 mg/mL) or low (0.5 mg/mL) concentration of polyLN521 and washed with PBS to reach a physiologic pH of about 7.4 to promote cell survival. Cells were loaded into the bioreactors at a density of 2.68 ± 0.97 × 10^8^ cells in 100 mL of StemFlex™ medium supplemented with ROCK inhibitor Y-27632 (10 μM). The initial rate of media perfusion was 0.1 mL/min and was gradually increased over 6 days in response to lactate levels, as per the manufacturer’s recommendations. When the predicted number of cells reached a plateau, cells were harvested, and samples were stained with Trypan blue and counted using a hemocytometer. The population-doubling level (PDL) was calculated using the following formula: PDL = (log N − log N0)/log, where N is the number of harvested cells, and N0 is the number of seeded cells [28].

### 2.12. Statistical Analysis

Data are shown as the mean ± standard deviation. Samples cultivated on basal-Matrigel, low-Matrigel, basal-LN521, low-LN521, basal-polyLN521, and low-polyLN521 were compared using a Student’s t-test or two-way analysis of variance (ANOVA). A *p*-value *p* < 0.05 was considered statistically significant. GraphPad Prism^®^ software, version 9.3.1 (GraphPad Software Inc., La Jolla, CA, USA), was used for statistical analyses.

## 3. Results

**Figure 1:**
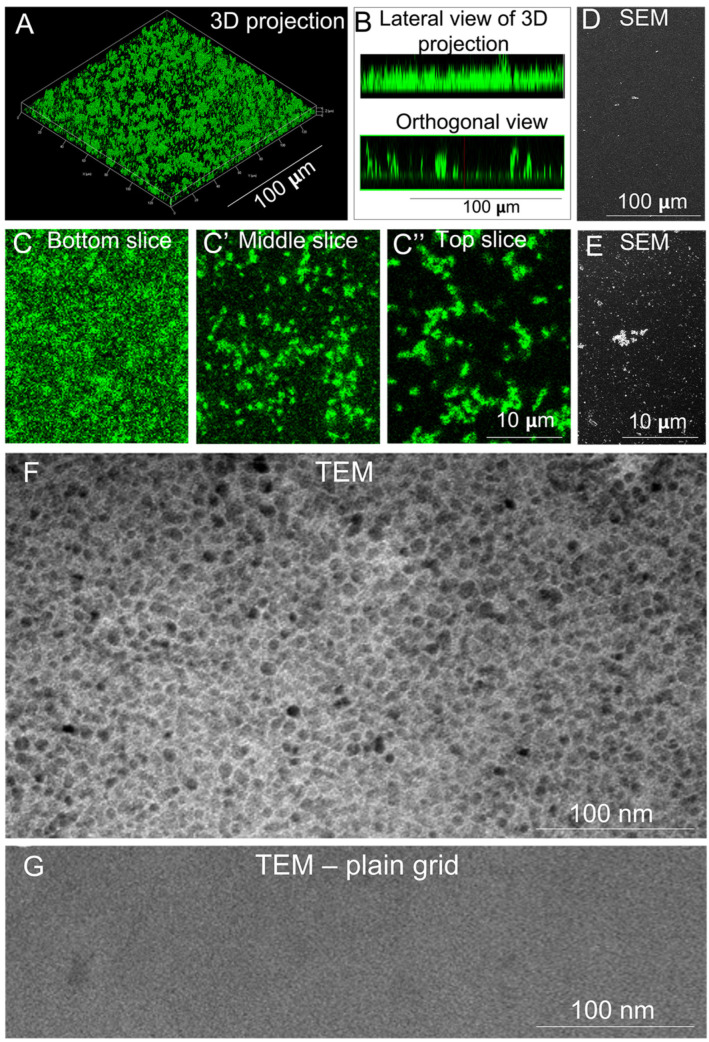
Structural analyses of polylaminin 521 (polyLN521). (**A**–**C**) The structure of polyLN521 is shown at progressively increasing magnifications from top to bottom rows, using laser scanning confocal fluorescence microscopy, scanning electronic microscopy (SEM,) and transmission electron microscopy (TEM). (**A**) A z-stack of confocal images is depicted after 3D reconstruction using the “surface” option of the ZEN software. B. Side and orthogonal views of the stacks are also shown. Panels (**C**–**C’’**) correspond to 3 optical slices selected to reveal the structure of polyLN521 at the bottom (**C**), middle (**C’**), and top (**C’’**) of the z-stack at higher magnification. (**D**,**E**) SEM images of polyLM521 at two magnifications. F-G. TEM images after the negative staining of polyLN521 (**F**) and of the plain copper grid that supports the sample (**G**). The polygonal-like pattern observed in (**F**) corresponds to the supramolecular array of the protein and not to the mesh of the grid support.

EHS LN and recombinant LN111 assemble in flat, homogeneous, polygonal-shaped layers [23,29]. We investigated whether polyLN521 displayed similar properties. The 3D projection of a z-stack obtained using confocal fluorescence microscopy revealed the formation of a homogeneous layer; however, no polygonal pattern was observed (Figure 1A,B, top). The orthogonal view of the stack showed protein clumps protruding from the surface (Figure 1B, bottom). The presence of these clumps was confirmed at a higher magnification (Figure 1C–C’’) and with SEM analysis (Figure 1D,E), but, again, no polygonal pattern was seen. We next examined polyLN521 with TEM to assess the structure of the protein layer on a nanometric scale. As shown in Figure 1F, polyLN521 displayed a homogeneous hexagonal-like structure similar to that assembled by EHS LN [22] and produced by cells [30]. To rule out the possibility that the supporting grid contributed to the observed pattern, we examined an image of the plain grid (Figure 1G). Together, these results demonstrate that, like EHS LN and LN111 polymerized at an acidic pH, polyLN521 assembles into a homogeneous polygonal-like layer, which makes it a superior substrate for cell culture.

**Figure 2:**
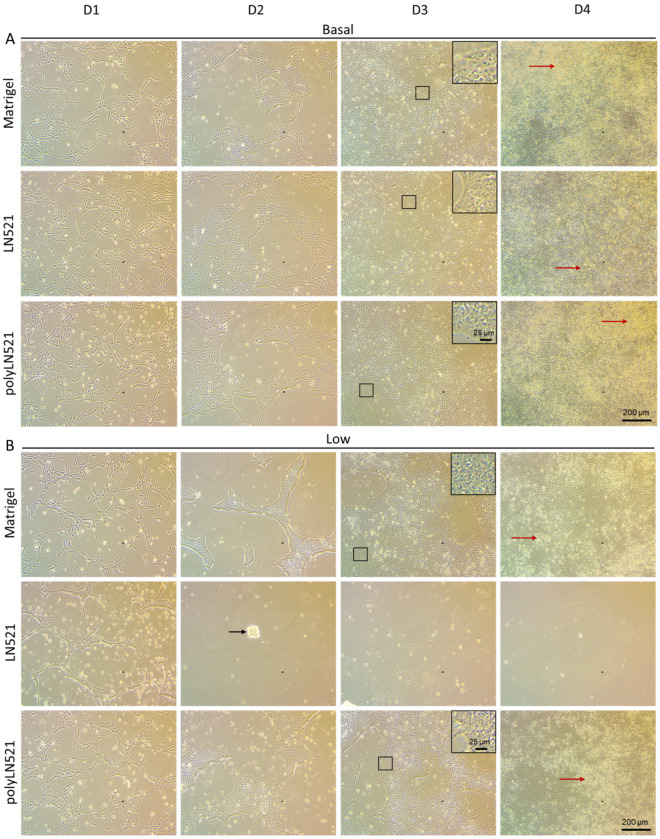
Expansion of human induced pluripotent stem cells (hiPSCs) under different coating conditions. (**A**) Representative bright-field images showing hiPSCs cultivated on basal-Matrigel, basal-LN521, and basal-polyLN521 from day 1 to day 4. (**B**) Representative bright-field images showing hiPSCs cultivated on low-Matrigel, low-LN521, and low-polyLN521 from day 1 to day 4. Insets represent cells with high nuclei to cytoplasm ratios. Red arrows indicate healthy colonies. Black arrow indicates dying cells.

**Figure 3:**
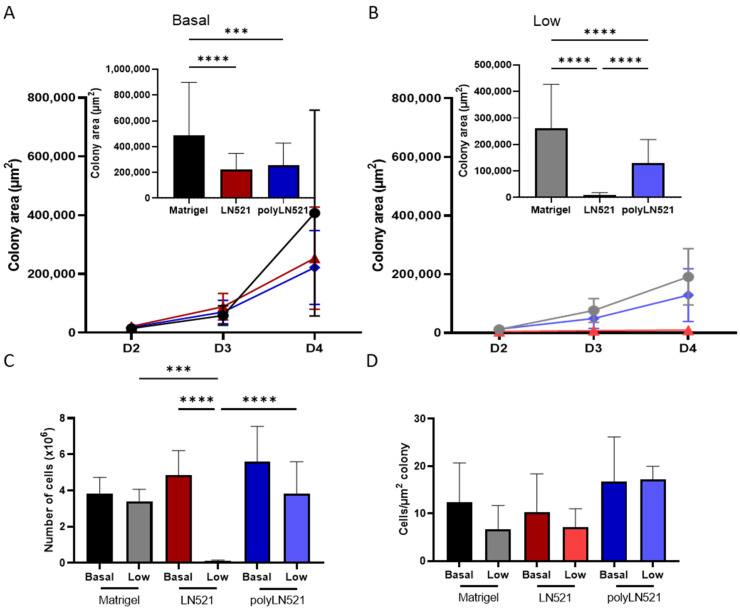
Quantification of expanded human induced pluripotent stem cells (hiPSC). (**A**) Colony area of basal-Matrigel, basal-LN521, and basal-polyLN521 at days 2, 3 and 4 of expansion. Bar graph shows the colony area on day 4 (Matrigel, *n* = 41; LN521, *n* = 47; polyLN521, *n* = 44). (**B**) Colony area of low-Matrigel, low-LN521, and low-polyLN521 at days 2, 3 and 4. Bar graph shows the colony area on day 4 (Matrigel, *n* = 40; LN521, *n* = 62; polyLN521, *n* = 91). (**C**) Number of cells harvested on day 4 (basal- and low-Matrigel, *n* = 9; basal-LN521, *n* = 9; low-LN521, *n* = 7; basal and low-polyLN521, *n* = 9). (**D**) Ratio of the number of cells to the colony area (basal- and low-Matrigel, *n* = 9; basal-LN521, *n* = 9; low-LN521, *n* = 7; basal- and low-polyLN521, *n* = 9). *** *p* < 0.001; **** *p* < 0.0001.

To further demonstrate that polyLN521 is a robust coating system, we cultivated two different hiPSC lines on basal or low concentrations of Matrigel, LN521, or polyLN521 (Figure 2 and Figure 3 and Supplementary Figure S1). At basal concentrations, all coating systems promoted cell attachment and proliferation (Figure 2A and Figure 3), and the cells exhibited pluripotent morphology. Round cells with a large nucleus to cytoplasm ratio were growing in young to mature colonies from day 1 to 4 (Figure 2A and Figure 3A) without spaces between them (Figure 2A, inset). On day 4, bright and compact colonies were observed (Figure 2A, red arrow). Although larger colonies were observed on basal-Matrigel (Figure 3A), no difference in cell number was seen between groups at the end of the expansion period (Matrigel: 3.84 × 10^6^ ± 0.87 × 10^6^; LN521: 4.85 × 10^6^ ± 1.35 × 10^6^; polyLN521: 5.58 × 10^6^ ± 1.95 × 10^6^) (Figure 3C), suggesting that the colonies grown on basal-LN521 and basal-polyLN521 were more compacted than those grown on basal-Matrigel.

At a low concentration, only polyLN521 was able to effectively support cell attachment and proliferation (Figure 2B and Figure 3B,C). Low-LN521 was unable to provide enough support for viable colonies to persist (Figure 2B, black arrow). This response was also observed when a second cell line was cultivated on a low concentration of LN521 and polyLN521 substrate (Supplementary Figure S1). On low-LN521, cells died at day 2 of expansion (Figure 2B and Supplementary Figure S1A), and no cell expansion was observed on low-LN521 at day 4 (0.1 × 10^6^ ± 0.05 × 10^6^) (Figure 2B and Figure 3B,C), in contrast to low-Matrigel (3.39 × 10^6^ ± 0.67 × 10^6^) or low-polyLN521 (3.81 × 10^6^ ± 1.77 × 10^6^). Similar morphologic parameters and cell numbers were observed when cells were cultivated on basal or low concentrations of Matrigel or polyLN521 (Figure 2 and Figure 3).

When we compared cell number and colony size on day 4 of expansion among the three coating systems for one of the hiPSC lines (SCVI20), we observed no differences, regardless of basal or low concentration (Figure 3D). In the other hiPSC line (SCVI274), however, we did observe differences in the ratio of cells per µm2 of colony between low-polyLN521 (23.57 ± 12.81) substrate and basal-Matrigel (5.57 ± 2.33), low-Matrigel (5.61 ± 1.05), and basal-LN521 (5.26 ± 4.04) substrates (Supplementary Figure S1E), although we attributed this difference to smaller colony size (132.63 ± 126.68 cm^2^) and fewer cells counted (2.52 × 10^6^ ± 0.80 × 10^6^). According to the morphologic parameters and proliferation rates, our data suggest that the polymerization of LN521 is crucial to supporting cell survival when using a lower concentration of coating system compared with the gold standard condition (basal-Matrigel).

**Figure 4:**
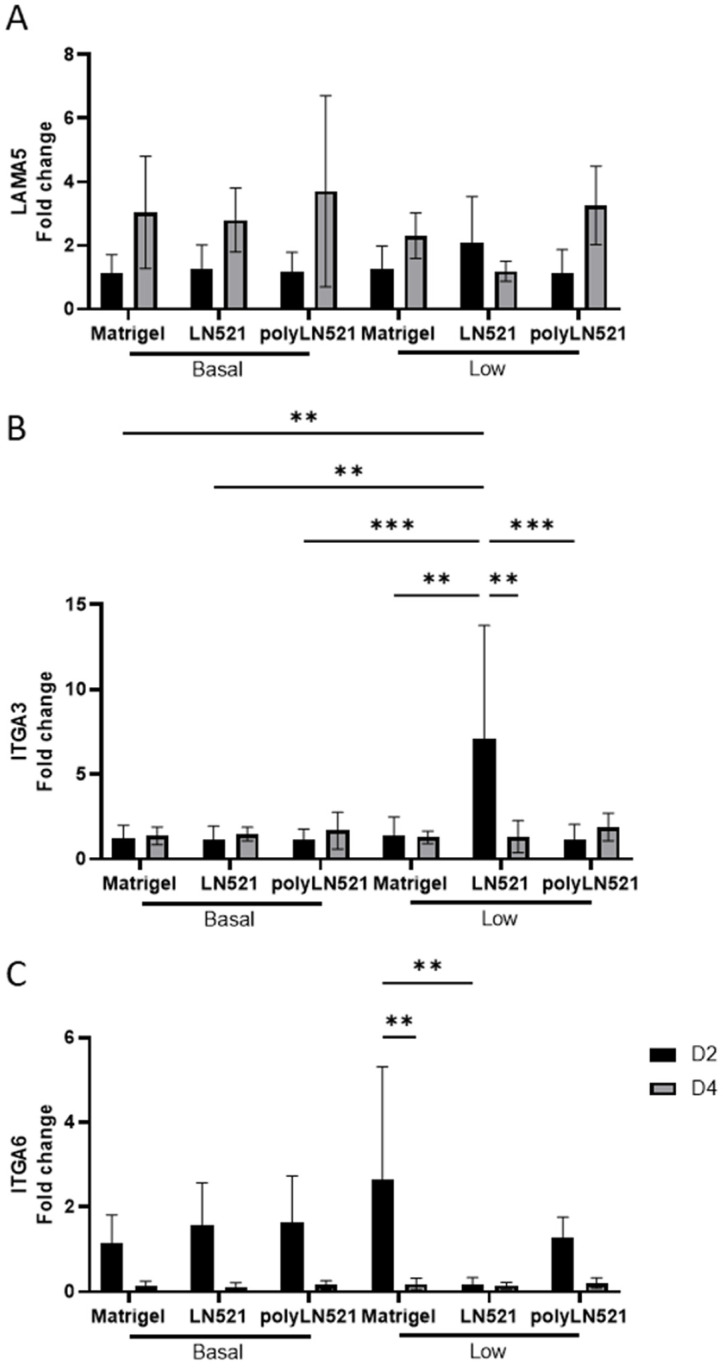
Gene expression indicative of extracellular matrix–cell interactions on days 2 and 4 in human induced pluripotent stem cells (hiPSCs) cultivated on coating systems with basal and low concentrations of Matrigel, LN521, or polyLN521. (**A**) Expression of LAMA5. (**B**) Expression of ITGA3. (**C**) Expression of ITGA6. *n* = 6 at day 2, *n* = 5 at day 4. ** *p* < 0.01; *** *p* < 0.001.

Among all coating conditions at basal concentrations, the expression of laminin alpha 5 (LAMA5), integrin alpha 3 (ITGA3), and integrin alpha 6 (ITGA6) was not significantly different at day 2 or day 4 of expansion (Figure 4). This trend was also seen for LAMA5 when the coating concentration was low (Figure 4A). However, the expression of ITGA3 increased significantly in cells cultured on low-LN521 at day 2 versus cells cultured on all other coating conditions at day 2 (basal and low concentrations) (Figure 4B). This increase in ITGA3 on low-LN521 at day 2 was followed by a significant decrease in expression at day 4. In addition, ITGA6 expression (Figure 4C) was increased in cells cultured on low-Matrigel at day 2 compared with day 4 and cells cultured on low-LN521 at day 2. No difference was observed when comparing Matrigel and polyLN521 at basal or low concentrations for any genes tested (Figure 4).

**Figure 5:**
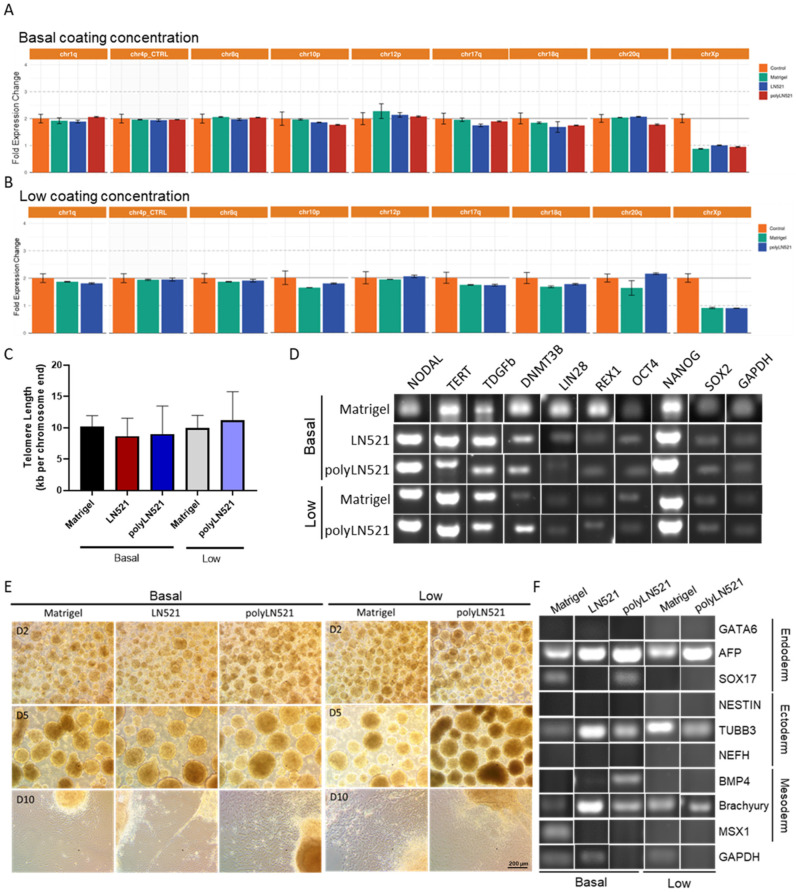
Characterization of pluripotency in human induced pluripotent stem cells (hiPSCs) cultivated on polyLN521. (**A**,**B**) Genetic screening analysis: quantitative PCR (qPCR) for Chr 1q, Chr 4p, Chr 8q, Chr 10p, Chr 12p, Chr 18q, Chr 20q and Chr Xp from cells cultivated on basal-Matrigel, basal-LN521, and basal-polyLN521 ((**A**), *n* = 3) or low-Matrigel, low-LN521, and low-polyLN521 ((**B**), *n* = 3). (**C**) Telomere length of hiPSCs (*n* = 4). (**D**) Pluripotent gene expression in hiPSCs. (**E**) Representative bright-field images showing the spontaneous differentiation of iPSCs on day 2, 5 and 10 after the cells were cultivated on basal- or low-Matrigel, basal- or low-LN521 and basal- or low-polyLN521. (**F**) Gene expression in the endoderm, ectoderm, and mesoderm of hiPSCs cultivated on basal- or low-Matrigel, basal- or low-LN521 and basal- or low-polyLN521.

After at least 3 cell passages on basal-Matrigel, basal-LN521, and basal-polyLN521, we did not detect any common karyotypic abnormalities previously reported in hiPSCs (Figure 5A,B). Among cell cultivated on all coatings and concentrations, telomere length was similar (basal-Matrigel: 10.22 ± 1.71, basal-LN521: 8.65 ± 2.88, basal-polyLN521: 9.01 ± 4.45, low-Matrigel: 9.97 ± 2.01, low-polyLN521: 11.22 ± 4.55; Figure 5C), and the pluripotent state of cells was maintained (Figure 5D, Supplementary Figure S2). In cells cultivated on basal and low concentrations of all coatings, we detected the expression of major hiPSC pluripotent genes (NODAL, TERT, DPPA4, DNMT3b, LIN28, REX1, NANOG, OCT4 and SOX2) (Figure 5D) and the high expression of OCT4, SOX2, NANOG, TRA1-60, and SSEA4 by using flow cytometry (Supplementary Figure S2).

Using a 10-day protocol, cells were induced to undergo spontaneous differentiation into the three embryonic germ layers on basal-Matrigel, basal-LN521, and basal-polyLN521 and on low-Matrigel and low-polyLN521 (Figure 5E,F). We observed small, rounded EBs on day 2 (Figure 5E, top); by day 5, EBs were larger and had well-defined borders (Figure 5E, middle). After the EBs were maintained in adherent cultures for an additional 5 days, cells with different morphologies spread from the EBs throughout the plate (Figure 5E, bottom). Furthermore, we detected the expression of at least one ectoderm gene (NESTIN and TUBB), one mesoderm gene (BMP4, brachyury (T), MSX1), and one endoderm gene (GATA6, AFP, SOX17) in cells at the end of spontaneous differentiation on Matrigel, LN521, and polyLN521 substrates (Figure 5F).

**Figure 6:**
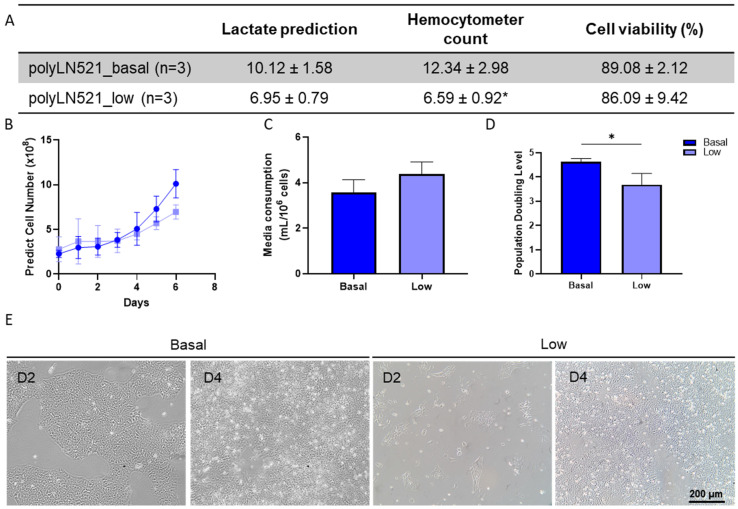
Large-scale expansion of human induced pluripotent stem cells (hiPSCs) using polyLN521. (**A**) Comparison between lactate-predicted and hemocytometer-counted cells harvested from the quantum expansion system (QES) after 6 days of expansion (data expressed in millions of cells). * *p* < 0.05, hemocytometer count for basal-polyLN521 vs. low-polyLN521. (**B**–**D**) Large-scale expansion characterization showing the lactate-predicted cell number (**B**), media consumption (**C**) and population-doubling level (**D**) of hiPSCs during expansion in QES on basal- and low-polyLN521 substrates. (**E**) Bright-field images of hiPSCs cultivated on basal-polyLN521 and low-polyLN521 after harvesting from the QES. * *p* < 0.05.

The large-scale expansion of hiPSCs using polyLN521 as a coating substrate was performed using the QES as previously described for LN521 [16]. We observed the exponential growth of hiPSCs, starting with 2.26 × 10^8^ ± 0.41 × 10^8^ and 2.89 × 10^8^ ± 1.26 × 10^8^ cells cultivated on basal-polyLN521 and low-polyLN521 that were expanded to 12.34 × 10^8^ ± 2.98 × 10^8^ and 6.59 × 10^8^ ± 0.92 × 10^8^ cells when harvested after 6 days, respectively (Figure 6A,B). Therefore, the large-scale expansion of hiPSCs on the polyLN521 coating system at either the basal or low concentration generated cells with high viability (>85%, Figure 6A) after 6 days of expansion. Although the cells expanded on low-polyLN521 did not reach the same yield compared with those expanded on basal-polyLN521, hiPSCs required a similar volume of medium per number of cells (3.57 ± 0.55 mL/10^6^ cells vs. 4.38 ± 0.52 mL/10^6^ cells, Figure 6C). In addition, hiPSCs had a significantly higher PDL when cultivated on the basal-polyL521 substrate than when cultivated on the low-polyL521 substrate (4.61 ± 0.14 vs. 3.68 ± 0.46, Figure 6D). Although hiPSCs cultivated in the QES on low-polyLN521 had a lower expansion rate, they attached and maintained a typical PSC morphology similar to hiPSCs grown on basal-polyLN521 (Figure 6E).

## 4. Discussion

To maintain an undifferentiated state and capability for self-renewal within their physiologic niche, stem cells require contact with an immobilized environment consisting of a thin layer of ECM, which can be mimicked by coating substrates in vitro. Traditional protein coating approaches, such as physical adsorption, require a non-physiologic, high protein concentration (>50 µg/mL) [31] to assure efficient hiPSC expansion [17]. Here, we described a cost-effective low concentration (0.5 µg/mL) polyLN521-based coating system capable of providing the homogeneous protein distribution essential for mimicking the in vivo environment of the niche [32,33] without the need of any additional coating components. This coating system supported the healthy expansion of hiPSCs, which was demonstrated by the presence of a classical colony morphology, the expression of pluripotent markers, the absence of karyotype abnormalities, and the capacity to spontaneously differentiate into the three germ lineages.

In initial studies of LN521 as a coating system, LN521 was applied at a very high concentration (30 µg/mL) to ensure iPSC support, proliferation, and stability [34]. With the advancement of techniques for purifying recombinant LNs and the production of more stable, defined media, the concentration of LN521 required for hiPSC generation and expansion has been decreased by 5 to 6 times [35,36]. In the present study, we demonstrated with 2 different hiPSC lines that cells did not proliferate or survive in vitro when LN521 was applied below the optimal concentration of 5 µg/mL. Similar results have been previously described by another group that showed small colonies and unstable cell morphology when culture dishes were coated with a combination of vitronectin (5 µg/mL) and LN511 or LN521 at concentrations below 0.5 µg/mL [19]. However, we have shown that, unlike LN521, polyLN521 supported hiPSC survival and expansion at a low concentration of 0.5 µg/mL. When the concentration of Matrigel was reduced by 10 times, we observed a trend of fewer cells per colony area, although this difference did not reach statistical significance. Furthermore, this trend disappeared when polyLN521 was used, suggesting the presence of more compact colonies after 4 days of expansion on polyLN521 substrate, at basal and low concentrations.

A different approach to cultivating hiPSCs under chemically defined conditions is the use of synthetic substrates such as polyvinyl alcohol (PVA), poly [poly (ethylene glycol) monomethacrylate] (PEGMA), and poly (N-isopropylacrylamide) (PNIPAM) [37,38]. Although synthetic polymers can be broadly applied on different surfaces and used to demonstrate that cells can be expanded and maintain their pluripotent characteristics, there are several technical limitations to this approach. Not only are multiple steps necessary to create the polymer [37], but an additional matrix is required to coat the system [39]. Furthermore, translating this approach to large-scale proportions is difficult [40], making the reproducibility of this system a bottleneck for those in the field. On the other hand, polyLN is an easy, chemically-defined, and stable system, facilitating reproducibility and a high-quality cell product, even when used at a low concentration.

The interaction between microenvironmental signals and intrinsic cell cues modulates hPSC self-renewal and differentiation [41]. When Matrigel or LN511 is used, hPSCs predominantly adhere to the substrate via ITGA5 and ITGA6 and proliferate via β1 integrin [42,43]. In addition, when cells were cultivated on vitronectin substrate [38], ITGA3 expression correlated with cell attachment and migration, suggesting that the substrate affects these in vitro interactions. Given that polyLN521 assembles in homogeneous layers, we evaluated the effect of coating concentration on the cell expression of LAMA5 and integrins (i.e., ITGA3 and ITGA6) on day 2 and day 4 of expansion. Our results suggested that the increased expression of ITGA3 in cells cultivated on low-LN521 substrate and a trend of increased expression of ITGA6 in cells cultivated on low-Matrigel substrate were compensatory responses to maintaining the attachment of cells to the substrate. Notably, the same response was not observed when cells were cultivated on low-polyLN521. Once the low-polyLN521 coating was spatially distributed, it recruited the same level of ITGA6 as observed on basal-poly521.

Cell morphology, karyotypic stability, pluripotency, and ability to differentiate spontaneously into ectoderm, mesoderm, and endoderm are the gold-standard characteristics of any PSC [44]. Recently, the chromatin state has been used as an indicator for pluripotent plasticity [45], whereby telomere length in the range of 9 to 12 kb has been used as an additional parameter for characterizing the undifferentiated state of cells [45,46,47]. Our results show that cells cultivated on polyLN521 effectively maintained their pluripotent state and exhibited the gold-standard characteristics of bright and compact colonies, a large nucleus to cytoplasm ratio, no karyotypic abnormalities, expression of pluripotent markers, telomere length in the range of 10 kb, and spontaneous differentiation into three germ layers. Although other groups reported a loss of plasticity in cells cultivated below the optimal dose of coating, [3,19] our results suggest that a low polyLN521 concentration did not impair cell characteristics.

In our previous study, we demonstrated that LN521 was an efficient coating substrate for the large-scale expansion of hiPSCs in a closed-cell expansion system [16,27]. However, LN521 is an expensive cell culture substratum, limiting the cost-effectiveness of this system. Therefore, optimization of the expansion protocol is imperative. To achieve this, polyLN521 was used to demonstrate that, with a low substrate concentration, cells survived, attached, and expanded in the closed large-scale cell expansion system, reaching cell numbers similar to those achieved when basal-LN521 was used. The superiority of basal-polyLN521 was also notable for its higher cell expansion efficiency (PDL, *p* < 0.05) than low-polyLN521. According to our previous report, a basal dose of polyLN521 generated 1.54-fold more cells after large-scale expansion than did basal-LN521 [16].

Our findings may have biotechnological implications that extend beyond the stem cell setting. Laminin is the major signaling component of all basement membranes in the body. This sheet-like type of extracellular matrix is responsible for providing structural and physiologic support to cells in several tissues such as the epithelial lining, endothelium, glands, muscles, and nerves. In particular, laminins containing the α5 subunit, such as LN511 and LN521, are ubiquitous components of stem cell niches [48,49]. The finding that polyLN521 displays the nanometric polygonal-like structure of laminin in natural basement membranes opens the possibility of its use to produce biomimetic lining for bioengineered tissues in the future. We have recently shown that substrates based on polyLM111 and polyLM511 induce the formation of biomimetic layers of stratified keratinocytes and tightly adhered endothelial cells [23].

## 5. Conclusions

PolyLN521 supported the highly efficient expansion of hiPSCs, even at a low substrate concentration. The ability of polyLN521 to support robust hiPSC growth can be attributed to its innate hexagonal-like structure, as demonstrated by the optimal distribution of its polymeric coating that was retained upon dilution. Cells cultivated on a lower concentration of polyLN521 substrate maintained their pluripotent phenotype and presented morphologic and molecular characteristics similar to those of cells grown on the gold-standard Matrigel substrate. The large-scale expansion of cells on basal and low concentrations of polyLN521 was feasible and cost effective. Thus, polyLN521 is an ideal candidate for a chemically-defined, xeno-free, and robust coating substrate for the production of large-scale batches of hiPSCs.

## Data Availability

The data that support the findings of this study are available from the corresponding author upon reasonable request.

## Supplementary Materials

The following supporting information can be downloaded at: https://www.mdpi.com/article/10.3390/cells11243955/s1, Figure S1: Expansion and quantification of a second human induced pluripotent stem cell (hiPSC) line (SCVI274) under different coating conditions; Figure S2: Analysis of protein expression in human induced pluripotent stem cells (hiPSCs) using flow cytometry; Table S1: List of primers used for cell characterization.
