# Supplementary material for: Polymerized Laminin-521: A Feasible Substrate for Expanding Induced Pluripotent Stem Cells at a Low Protein Concentration
Source: Cells. 2022 Dec 7;11(24):3955. doi: 10.3390/cells11243955 (PMC9777247; doi:10.3390/cells11243955)
Supplement: Supplementary file 1 [file cells-11-03955-s001.zip › cells-2026749-supplementary.pdf]

Supplementary Figures

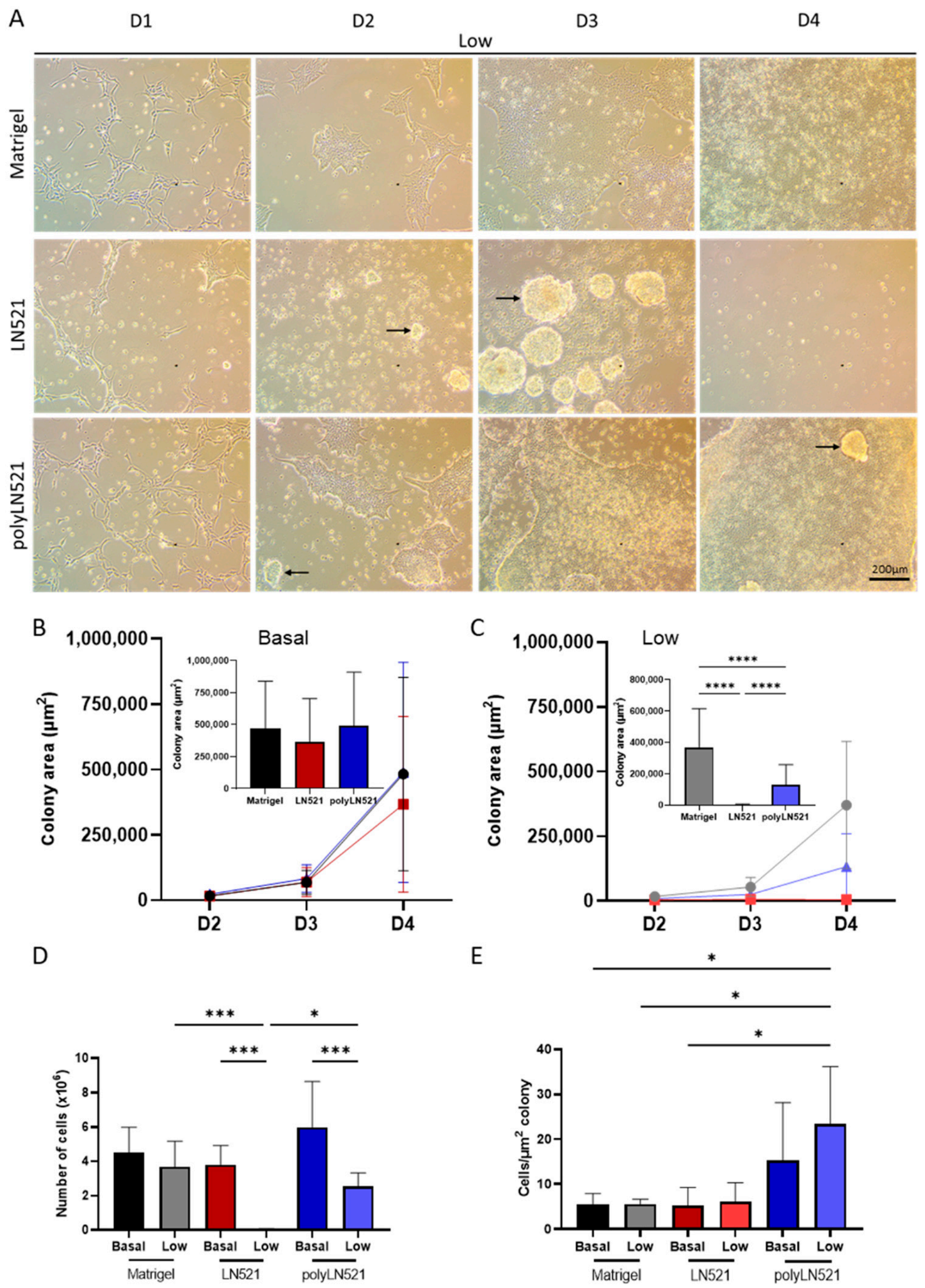

Figure S1. Expansion and quantification of a second human induced pluripotent stem cell (hiPSC) line (SCVI274) under different coating conditions. (A) Representative bright-field images showing hiPSCs cultivated on low-Matrigel, low-LN521, or low-polyLN521 from day 1

to day 4 (D1-D4). Black arrows indicate dying cells. (B) Colony area on days 2, 3, and 4 after hiPSCs were cultivated on basal-Matrigel, basal-LN521, or basal-polyLN521. Bar graph shows colony area on day 4 (Matrigel, n=29; LN521, n=33; polyLN521, n=16). (C) Colony area on days 2, 3, and 4 after hiPSCs were cultivated on low-Matrigel, low-LN521, or low-polyLN521. Bar graph shows colony area on day 4 (Matrigel, n=30; LN521, n=44; polyLN521, n=108). (D) Number of cells harvested on day 4 (basal- or low-Matrigel, n=9; basal-LN521, n=8; low-LN521, n=7; basal- or low-polyLN521, n=9). (E) Ratio of the number of cells to colony area (basal- or low-Matrigel, n=9; basal-LN521, n=9; low-LN521, n=8; basal- or low-polyLN521, n=9). *p<0.05, ***p<0.001; ****p<0.0001.

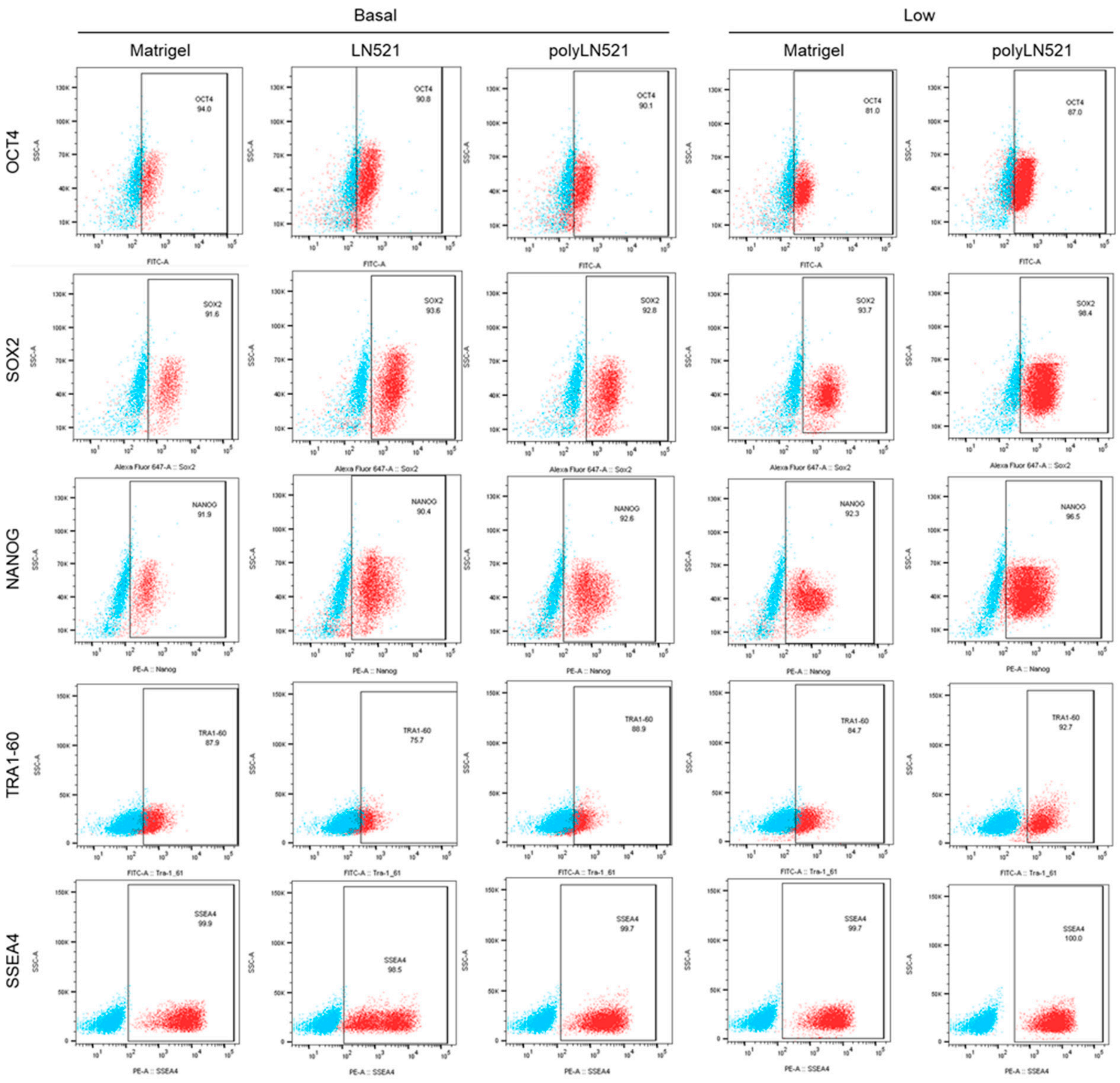

Figure S2. Analysis of protein expression in human induced pluripotent stem cells (hiPSCs) using flow cytometry. Representative dot plots showing *OCT4*, *SOX2*, *NANOG*, *TRA1-60* and, *SSEA4* expression in hiPSCs cultivated on basal- or low-Matrigel, basal-LN521, and basal- or low-polyLN521. Blue dots represent the isotype control.

Table S1. List of primers used for cell characterization.

	Primer	Forward	Reverse
Pluripotency	OCT4	AGCCTGAGGGCGAAGCAGGA	CCCCAGGGTGAGCCCCACAT
	NANOG	CAGCCCTGATTCTTCCACCAGTCCC	TGGAAGGTTCCCAGTCGGGTTCACC
	LIN28	CAAAAGGAAAGAGCATGCAGAA	ATGATCTAGACCTCCAGAGTTGTAGC
	SOX2	GAGAAGTTTGAGCCCCAGG	AGAGGCAAACTGGAATCAGG
	REX1	CAGATCCTAAACAGCTCGCAGAAT	GCGTACGCAAATTAAAGTCCAGA
	TDGF1	CTGCTGCCTGAATGGGGGAACCTGC	GCCACGAGGTGCTCATCCATCACAAGG
	NODAL	GGGCAAGAGGCACCGTCGACATCA	GGGACTCGGTGGGGCTGGTAACGTTTC
	TERT3	CCTGCTCAAGCTGACTCGACACCGTG	GGAAAAGCTGGCCCTGGGGTGGAGC
	GDF3	CTTATGCTACGTAAAGGAGCTGGG	GTGCCAACCCAGGTCCCGGAAGTT
	DNMT3B	TGCTGCTCACAGGGCCCGATACTTC	TCCTTTCGAGCTCAGTGCACCACAAAAC
Ectoderm	NESTIN	CACCTCAAGATGTCCCTCAG	AGCAAAGATCCAAGACGCC
	TUBB3	GCTCAGGGGCCTTTGGACATCTCTT	TTTTCACACTCCTTCCGCACCACATC
	NEFH	ACCTATACCCGAATGCCTTCTT	AGAAGCACTTGGTTTTATTGCAC
Mesoderm	MSX1	CGAGAGGACCCCGTGGATGCAGAG	GGCGGCCATCTTCAGCTTCTCCAG
	BMP4	GCACTGGTCTTGAGTATCCTG	TGCTGAGGTTAAAGAGGAAACG
	Brachyury (T)	GCCCTCTCCCTCCCCTCCACGCACAG	CGGCGCCGTTGCTCACAGACCACAGG
Endoderm	GATA6	CCAACTGTCACACCACAAC	TGGGGGAAGTATTTTTGCTG
	AFP	GAATGCTGCAAACTGACCACGCTGGAAC	TGGCATTCAAGAGGGTTTTCAGTCTGGA
	SOX17	GACGACCAGAGCCAGACC	CGCCTCGCCCTTCACC
Cell-ECM	LAMA5	CAAGAGGGACTGTCTGGAGC	CCACGATCTCTCCGTTCTCC
	ITGA3	TGCCTACAACTGGAAAGGAAAC	CTGCCTACCTGCATCGTGTA
	ITGA6	CCTCCCTGAGCACATATTCG	CACCTCCAACTTCTCCATCTC
	GAPDH	AATCCCATCACCATCTTCCAG	AAATGAGCCCCAGCCTTC
